# Presence of Papillomavirus DNA sequences in the canine transmissible venereal tumor (CTVT)

**DOI:** 10.7717/peerj.7962

**Published:** 2019-10-25

**Authors:** Sergio Ayala-Díaz, Roberto Jiménez-Lima, Katia M. Ramírez-Alcántara, Marcela Lizano, Leonardo J. Castro-Muñoz, Diego O. Reyes-Hernández, Jaime Arroyo-Ledezma, Joaquín Manzo-Merino

**Affiliations:** 1 Universidad del Mar, Puerto Escondido, Oaxaca, Mexico; 2 Programa de Maestría y Doctorado en Producción y Sanidad Animal, Universidad del Mar, Puerto Escondido, Oaxaca, Mexico; 3 Clinical Research Division, Instituto Nacional de Cancerología, Mexico City, Mexico; 4 Basic Research Division, Instituto Nacional de Cancerología, Mexico City, Mexico; 5 Programa de Posgrado en Ciencias Biológicas, Universidad Nacional Autónoma de México, Mexico City, Mexico; 6 Departamento de Medicina Genómica y Toxicología Ambiental, Instituto de Investigaciones Biomédicas, UNAM, Mexico City, Mexico; 7 Programa de Posgrado en Ciencias Médicas Odontológicas y de la Salud, Universidad Nacional Autónoma de México, Mexico City, Mexico; 8 Asociación Esteriliza y Educa A.C., Puerto Escondido, Oaxaca, Mexico; 9 Cátedras CONACyT-Instituto Nacional de Cancerología, Mexico City, Mexico

**Keywords:** Canine transmissible venereal tumor, Papillomavirus, PCR

## Abstract

**Background:**

The canine transmissible venereal tumor (CTVT) or Sticker’s sarcoma is a neoplastic disease affecting dogs. This disease is presented as a tumoral mass in the genital organs of both, male and female individuals. Up to date, there is no clear evidence indicating a viral agent as the causative mediator for CTVT development.

**Purpose:**

The present work aims to analyze 21 samples from canines with CTVT for molecular identification of Papillomavirus DNA sequences. In addition, microbiological analysis, cytologic and histopathologic evaluations were also performed.

**Results:**

All patients showed no biochemical and microbiological alterations. Molecular analysis demonstrated the viral DNA presence in the samples using different primer sets. The MY primers amplified a 450 bp band in seven out of 21 samples (33%). The PVF and Fap64 primer set, targeting the L1 sequence of Canine Papillomavirus (CPV), showed positivity in 16 out of 21 samples (76%).

**Conclusion:**

These results support the possible causative association between CPV and CTVT; nevertheless, additional studies are required to uphold such statement. This work presents evidence indicating that a viral agent might be involved in the pathogenesis of CTVT and set the bases for a better understanding of the CTVT pathobiology.

## Introduction

The canine transmissible venereal tumor (CTVT), also known as infectious sarcoma, venereal granuloma, transmissible lymphosarcoma or Sticker’s sarcoma, is a neoplastic disease propagated typically during the intercourse in dogs. The symptoms mostly exhibiting an extra genital presentation. This neoplasm appears as a tumoral mass at the glans bulb in males, or in the vaginal vestibule in females (Ganguly, Das & Das, 2016), and normally has a high ratio of chemotherapy response (Birhan & Chanie, 2015). The CTVT is found more frequently at the genital area, while less in the oral cavity, nasal cavity, eyes or on the skin (Ganguly, Das & Das, 2016; Ojeda et al., 2016). The CTVT occurrence is worldwide, with the highest incidence in tropical areas. In Mexico, the CTVT is shown to be the most prevalent cancer type among stray dogs, with a rate as high as 34% in the analyzed cases (Ortega-Pacheco et al., 2003).

After implantation, tumor mass starts to grow after 15–60 days, initially displaying a slow and unpredictable behavior and eventually becoming malignant (Ganguly, Das & Das, 2016). Different approaches designed to study the genetic origin of the CTVT suggest that this type of tumors share a common ancestor that was spread in the dog population worldwide (Murgia et al., 2006; Strakova et al., 2016). Nevertheless, despite several studies focusing on the immunological behavior, chromosomal abnormalities and immunophenotype, the molecular mechanisms altered by CTVT cells to avoid the immune response remain unclear (González et al., 2002; Siddle & Kaufman, 2013). There is still a discrepancy among researchers regarding the causative agent of the CTVT.

Although some works have described the presence of particles similar to virus in this neoplasm, but the evidence indicating that a virus is the responsible for CTVT remains unclear. Among the different cancer-causing viruses, the Papillomavirus (PV) family is responsible for almost a 5% of human cancers (Plummer et al., 2016). In this regard, PV infect a plethora of species such as reptiles, birds and mammals, including humans and dogs (Rector & Van Ranst, 2013). Several diseases caused by PV in dogs include proliferative disorders such as oral papilloma, oral papillomatosis and pigmented plaques which are mostly self-resolving (Munday et al., 2016). Additionally, PV has been associated to the development of squamous cell carcinoma in dogs (Zaugg et al., 2005) and other animal species such as horses and cattle indicating a causative role of PV in neoplastic lesions (Carvajal et al., 2012; Díaz et al., 2012). Since the oncogenic potential of PV has been well established (Smith et al., 2016) and the presence of particles similar to viruses has been reported in CTVT, it is of special interest in testing the presence of PV sequences in the CTVT to study its possible association with the disease.

## Materials and Methods

### Type of study and ethics statement

This was a cross sectional study, carried out in Puerto Escondido, Oaxaca, Mexico. After reviewing and approval of the protocol by the Institutional Committee, a total of 21 specimens attended at the Clinical Oncology Service of the civil association Esteriliza & Educa A.C. were enrolled.

Inclusion criteria: patients with positive diagnosis of CTVT by clinical examination, confirmed by cytological and histological analysis, both sexes, at reproductive age (between 2 and 5 years old), who have not received previous chemotherapy treatment. The owner signed the informed consent and commitment letter.

The Asociación Esteriliza y Educa A.C. approved this study.

### Hemato-biochemical analysis

In order to assess the general condition of the patients in their first consultation, blood samples were collected from each patient for hemogram and clinical biochemistry using a Vacutainer^®^ system. Blood samples were analyzed using the Cell-Dyn 1800 device (ABBOT, Chicago, IL, USA) based on impedance resistance method. Then, a peripheral blood smear was performed and stained according to the Wright technique for differential and morphological analysis.

Levels of glucose, urea, BUN, creatinine, cholesterol, triglycerides and uric acid were obtained through the ChemWell^®^ 2910 equipment (Awareness technology, Palm City, FL, USA) following the manufacturer’s instructions.

### Pathological analysis

Clinical examination of the patients included hepatic, splenic and lymph node palpation. During clinical examination, one imprint was taken from the surface of the tumor mass for microbiological analysis as well as for Papanicolaou and Wright staining to confirm the clinical diagnosis. Additionally, one smear was taken for Gram staining. Likewise, incisional biopsies were taken, which were fragmented into two parts: one for the histopathological study of the tumor mass and one for DNA extraction, the latter was preserved in physiological saline solution at −20 °C until analysis. Simultaneously, two imprints were taken from the extracted tumor mass per patient.

Tissue samples were placed into sterile containers with Phosphate Buffered Saline/10% formaldehyde for processing, according to the standard technique; two to four μm tissue sections were made with a microtome and then stained with Hematoxylin and Eosin technique for subsequent visualization under an optical microscope, Primo Star (Carl Zeiss, San Diego, CA, USA).

### DNA extraction

DNA extraction from biopsies was performed using the DNeasy Blood and Tissue Kit (Qiagen, Germantown, MD, USA.) according to the manufacturer’s instructions. To verify the integrity of the resulting DNA, an electrophoresis was performed on a 1% agarose gel using 1× TAE buffer. Gel was visualized in the MultiDoc-It™ Imaging System (Ultra-Violet Products Ltd., Cambridge, UK) using Gel Red as a dye.

### PV sequences detection

Papillomavirus sequences were identified in biopsies using universal primers aimed to detect the L1 region of PV (MY09 and MY11) (Gravitt et al., 2000). Specific primers for Canine Papillomavirus (CPV), PVf and FAP64 for the L1 gene and CP4 and CP5 for the E1 gene, were used (Lange et al., 2011). DNA integrity was verified by amplifying the β-globin gene (primers: GH20 and PC04) or the enzyme glyceraldehyde-3-phosphate dehydrogenase (primers: dogGAPDHf and dogGAPDHr) following the protocol established by Luff et al. (2012). Table 1 describes the primer sequences used in this study. The amplification conditions are described in Table 2 for each pair of primers.

**Table 1:** Primers used for Papillomavirus detection and analysis of genetic integrity. Primers used in this study for Papillomavirus detection and analysis of genetic integrity are listed.

Primer	Sequence (5′-3′)	Sense	Product
MY09	CGTCCMARRGGAWACTGATC	Fwd	450 bp
MY11	GCMCAGGGWCATAAYAATGG	Rev	
PVf	CTTCCTGAWCCTAAYMAKTTTGC	Fwd	450 bp
FAP64	CCWATATCWVHCATNTCNCCATC	Rev	
CP4	ATGGTACARTGGGCATWTGA	Fwd	500 bp
CP5	GAGGYTGCAACCAAAAMTGRCT	Rev	
GH20	GAAGAGCCAAGGACAGGTAC	Fwd	268 bp
PC04	CAACTTCATCAACGTTCACC	Rev	
dogGAPDHf	GGTGATGCTGGTGCTGAGTA	Fwd	585 bp
dogGAPDHr	GACCACCTGGTCCTCAGTGT	Rev	

**Table 2:** Amplification protocols for PCR by primers set. Amplification conditions used in this study are indicated.

Primer	Denaturing	Annealing	Elongation	Cycles
MY09	94 °C/50 s	55 °C/50 s	72 °C/55 s	38
MY11				
PVf	94 °C/1 min	50 °C/1 min	72 °C/1 min	45
FAP64				
CP4	94 °C/30 s	42 °C/30 s	72 °C/30 s	40
CP5				
GH20	94 °C/50 s	55 °C/50 s	72 °C/55 s	38
PC04				
dogGAPDHf	94 °C/30 s	50 °C/30 s	72 °C/30 s	45
dogGAPDHr				

### Statistical analyses

Descriptive analysis was performed using mean as a central tendency measure, as well as absolute and relative frequencies.

## Results

### Population description

A total of 21 patients with a clinical diagnosis of CTVT were analyzed, of which seven (33%) were males and 14 (67%) females. Of individuals included, 20 (95%) were non-neutered and one (5%) castrated. After corroborating the clinical diagnosis by cytology and histopathology, all the 21 patients were enrolled in the study. All the enrolled patients exhibited presence of neoplasia in the reproductive system. The analyzed samples showed no alterations in the hemogram and biochemical values, fitting within biological reference intervals for the species (Nuñez Ochoa, 2019) (Table S1). All patients received chemotherapy treatment based on Vincristine Sulphate (Crivosin^®^ Vet; Pisa Agropecuaria, Guadalajara, Mexico) at a dose of 0.023 mg/Kg for five or six cycles weekly. After sampling each case, the patients were monitored for 3 months after receiving the last cycle of chemotherapy. No recurrence was observed in any of the 21 patients. Table 3 summarizes the clinical information of the patients.

**Table 3:** Population description. Demographic characteristics: sex, site of tumor, breed and type of treatment.

Patient	Sex	Breed	Reproductive status	Site of tumor	Treatment
1	M	Mixed	Non-neutered	Penis	Vincristine 5 cycles
2	M	Mixed	Non-neutered	Penis	Vincristine 5 cycles
3	F	Mixed	Non-neutered	Vestibulum	Vincristine 6 cycles
4	M	Mixed	Neutered	Penis	Vincristine 5 cycles
5	F	Mixed	Non-neutered	Vestibulum	Vincristine 5 cycles
6	F	Mixed	Non-neutered	Vestibulum	Vincristine 5 cycles
7	F	Mixed	Non-neutered	Vestibulum	Vincristine 5 cycles
8	F	Mixed	Non-neutered	Vestibulum	Vincristine 5 cycles
9	M	Mixed	Non-neutered	Penis	Vincristine 5 cycles
10	F	Mixed	Non-neutered	Vestibulum	Vincristine 6 cycles
11	M	Mixed	Non-neutered	Penis	Vincristine 5 cycles
12	F	Mixed	Non-neutered	Vestibulum	Vincristine 6 cycles
13	F	Mixed	Non-neutered	Vestibulum	Vincristine 6 cycles
14	F	Mixed	Non-neutered	Vestibulum	Vincristine 5 cycles
15	M	Mixed	Non-neutered	Penis	Vincristine 5 cycles
16	F	Mixed	Non-neutered	Vestibulum	Vincristine 6 cycles
17	F	Mixed	Non-neutered	Vestibulum	Vincristine 6 cycles
18	F	Mixed	Non-neutered	Vestibulum	Vincristine 5 cycles
19	M	Mixed	Non-neutered	Penis	Vincristine 6 cycles
20	F	Mixed	Non-neutered	Vestibulum	Vincristine 6 cycles
21	F	Mixed	Non-neutered	Vestibulum	Vincristine 5 cycles

### Macroscopic diagnosis

**Figure 1:**
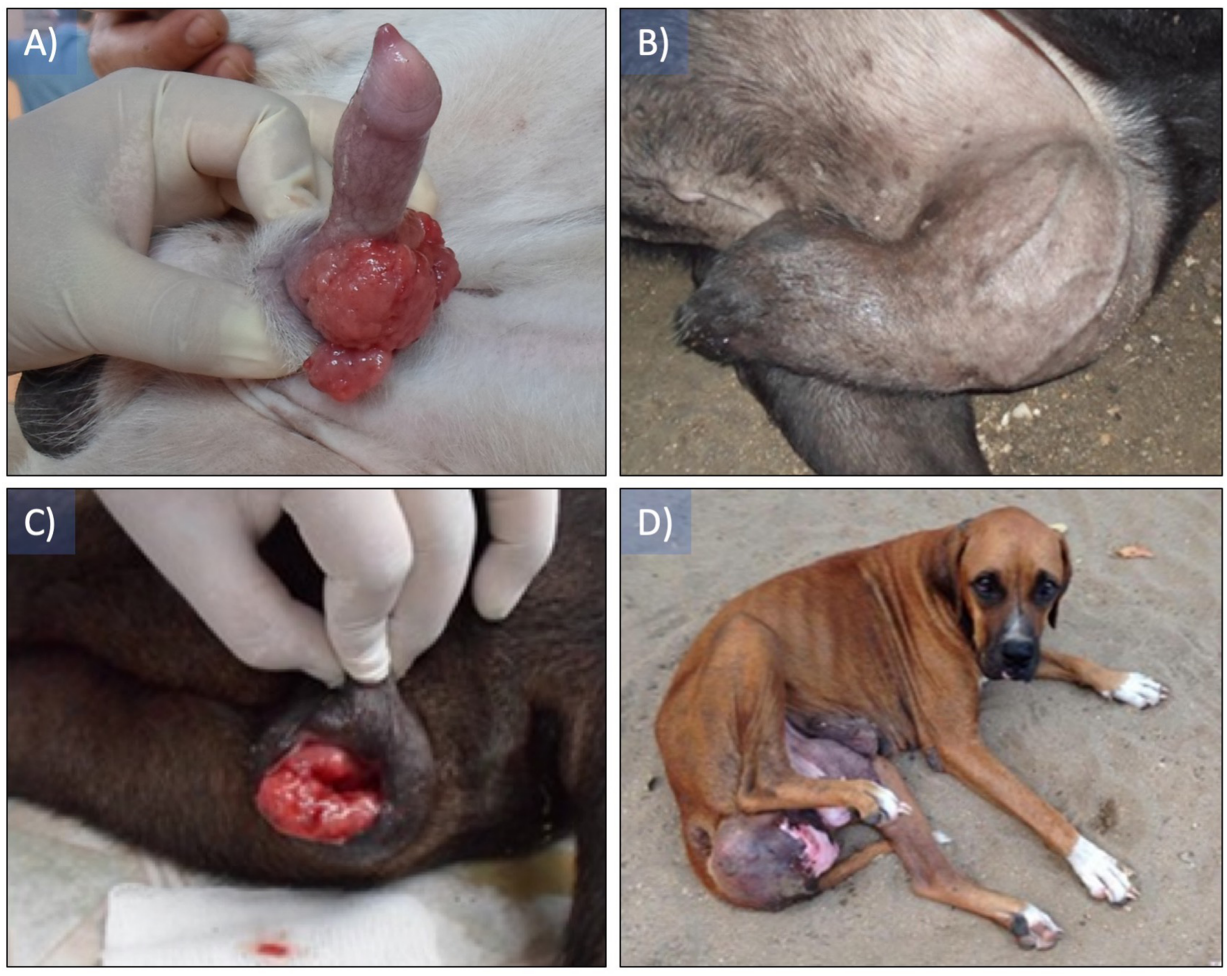
Canine transmissible venereal tumor presentation in patients. (A) The presence of a tumor mass can be observed in the dorsum and the sides of the bulb of the penis, (B) the tumor mass size impedes the correct drainage of the penis, (C) a tumor mass protrudes from the vulva, (D) tumor mass completely invading the vagina’s lumen.

Patients with a clinical diagnosis of CTVT displayed newly formed tissue of variable size, in the vestibule of the vagina in females and on the dorsum of the penile bulb in males, showing colorations ranging from pink to red, superficial and in some cases pedunculated, hemorrhagic, fragile, projecting toward the lumen of the vagina and the foreskin with diameters between 10 and 15 cm (Fig. 1).

### Microbiological analysis

The microbiological analysis of the samples indicated scarce colony-forming units of *Staphylococcus epidermidis* in the 28.6% of the patients, isolated as a single strain, which is considered as normal microbiota.

### Cytological analysis

**Figure 2:**
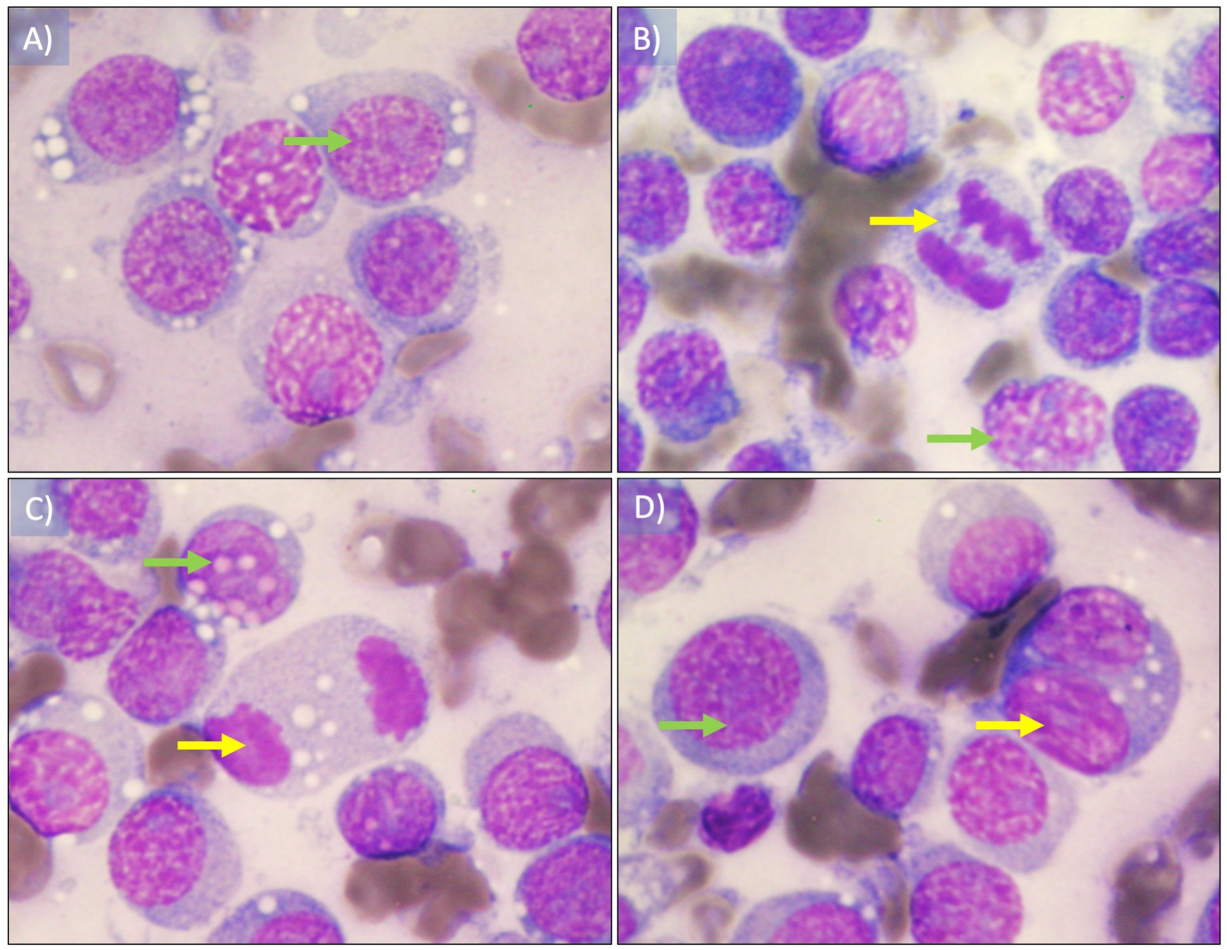
Cytological imprint of canine transmissible venereal tumor. (A) Red blood cells as well as round to ovoid cells with vacuolated eosinophilic cytoplasm are observed, also hyperchromatic round nucleus with one or two conspicuous nucleoli (green arrow). (B and D) mitotic figures observed in early and (C) later anaphase (yellow arrows). The plasmocitoid morphology tumor cells was 57% (continuous blue arrow) and the lymphocytoid morphology was 43% (blue dashed arrow), the nucleus-cytoplasm ratio was 4:1. Wright staining was used. Images were taken under a 100× magnification.

The presence of ovoid cells with vacuolated eosinophilic cytoplasm, hyperchromatic round nucleus with one or two conspicuous nucleoli was observed. Likewise, some atypical mitotic figures were observed, and the nucleus cytoplasm ratio was 4:1 (Fig. 2). The plasmocitoid 172 morphology tumor cells was 57% and the lymphocytoid morphology was 43%.

### Histopathological analysis

**Figure 3:**
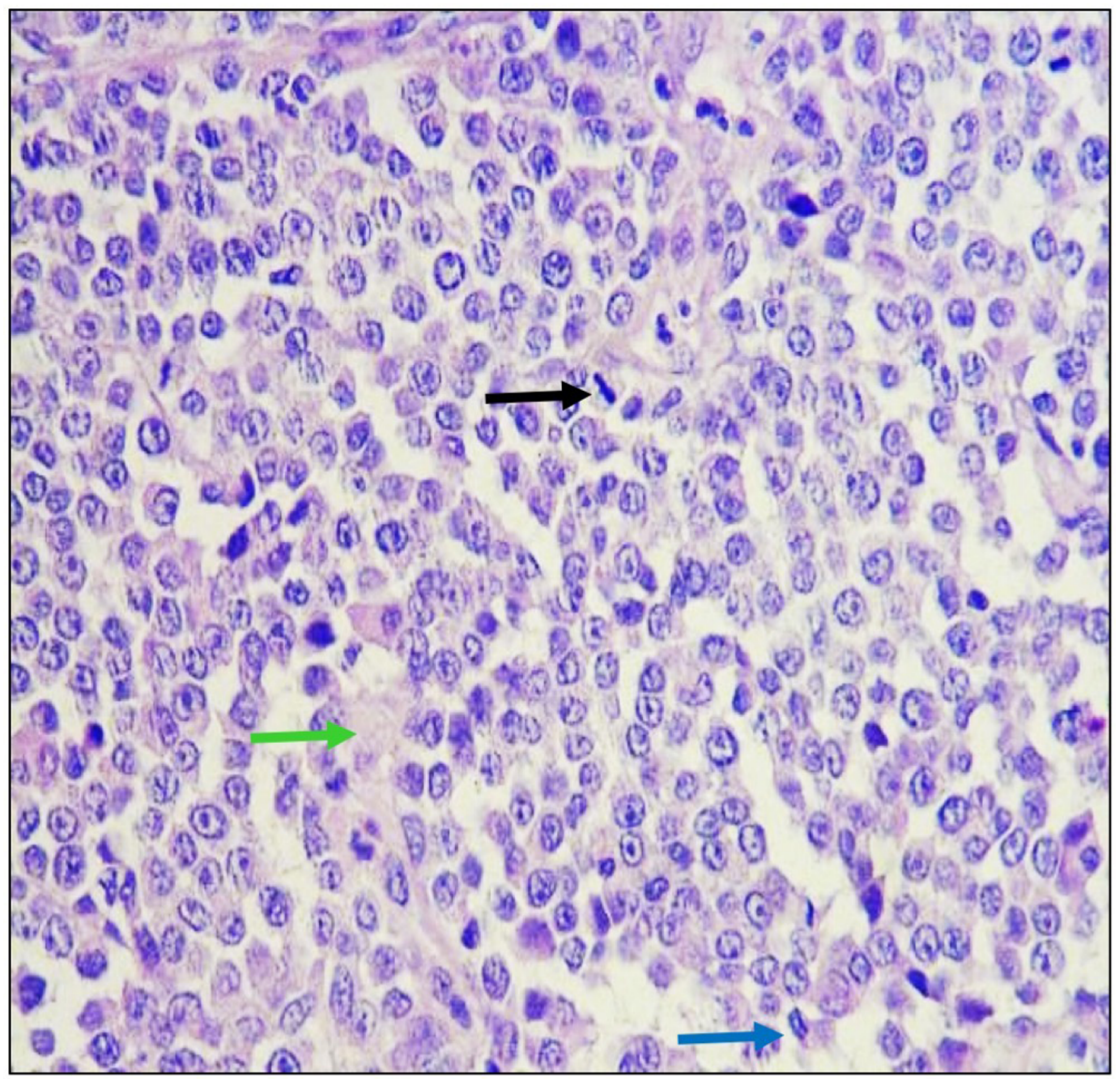
Histological analysis of biopsies. Tumoral tissue is observed composed of cells that are arranged in mantles and in a dispersed manner, which have scanty, ill-defined eosinophilic cytoplasm and oval nuclei with fine granular chromatin and conspicuous nucleoli, identifying mitosis in a dispersed pattern in metaphase (black arrow) and anaphase (blue arrow) mainly. The observed stroma is lax (green arrow) and there are congestive vessels with neoplastic cells on the wall. Hematoxilin and Eosin staining was used. Images were taken under a 40× magnification.

Formalin-fixed Parafin embeeded sections were stained with Hematoxylin and Eosin. Neoplastic lesion formed by cells were arranged in mantles and in a dispersed manner, presenting a poorly defined eosinophilic cytoplasm and nuclei ovals with fine granular chromatin and prominent nucleoli, numerous atypical mitoses were identified in a dispersed manner (Fig. 3). The stroma was lax and there were congestive vessels with neoplastic cells on the wall.

### Papillomavirus sequences are present in CTVT samples

**Figure 4:**
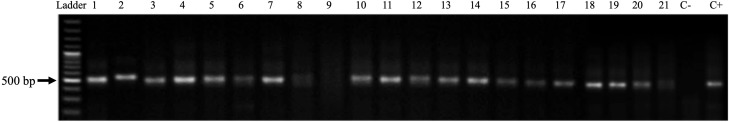
Amplification of the GAPDH enzyme gene from genomic DNA obtained from biopsies of CTVT. Ladder: Molecular Weight Marker, GeneRuler; lanes 1–21: biopsy samples; H_2_O: negative control and C+: HeLa DNA (positive control). In samples 1–8 and 10–21, a band corresponding to the amplicon of the GAPDH gene (586 bp) is observed.

**Figure 5:**
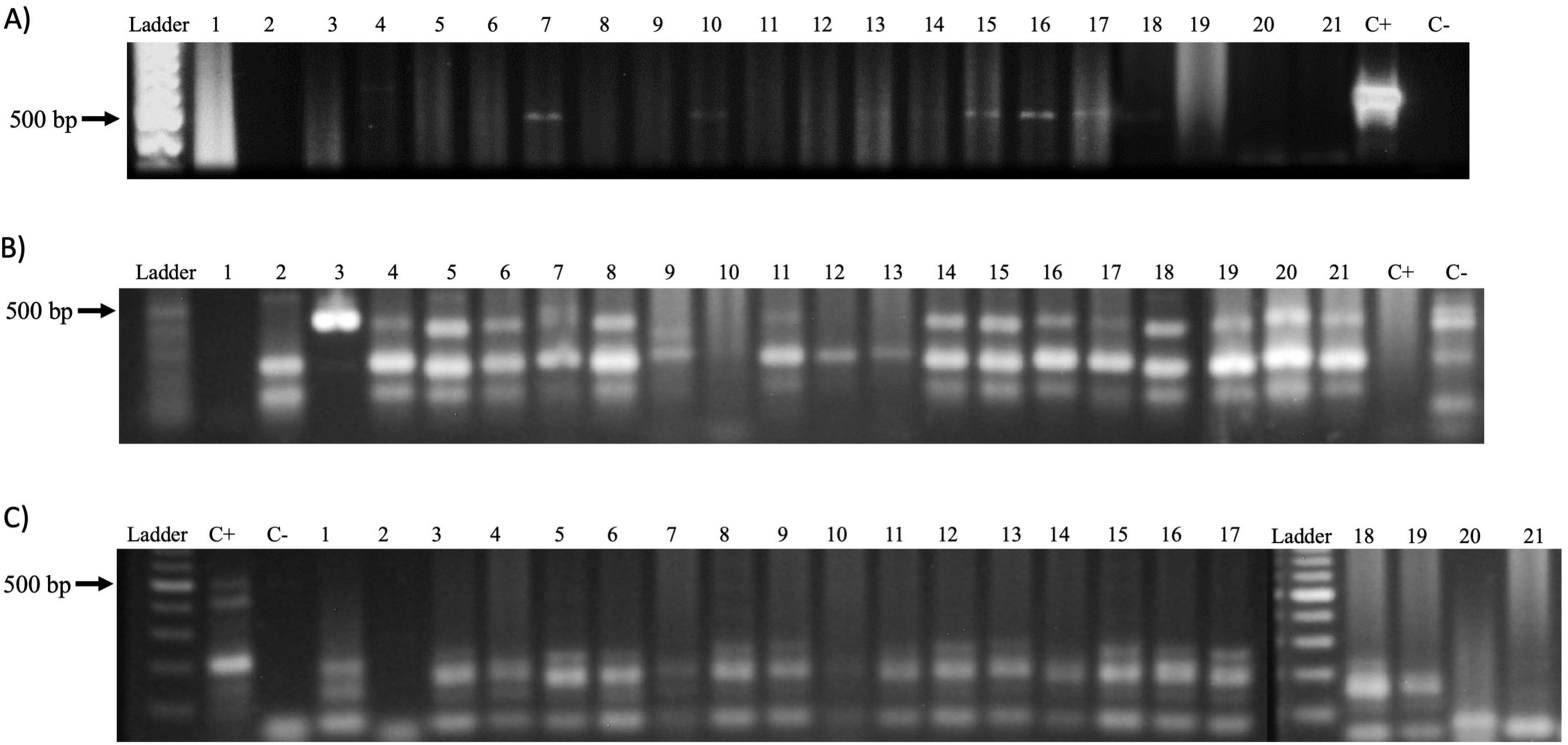
PV detection in samples. (A) Amplification of the Papillomavirus L1 gene with primers MY09 and MY11 from genomic DNA obtained from biopsies of CTVT. Ladder: Molecular Weight Marker, GeneRuler; lanes 1–21: biopsy samples; H_2_O: negative control and C+: HeLa DNA (positive control). Samples 7, 10, 13 and 14–17 a band corresponding to the amplicon of the L1 gene of Papillomavirus (450 bp) is observed. (B) Amplification of the L1 gene of Canine Papillomavirus with the primers PVF/FAP-64 from genomic DNA obtained from biopsies of CTVT. Ladder: Molecular Weight Marker, GeneRuler; H_2_O: negative control and C+: HeLa DNA (positive control); lane 1–21: biopsy samples. A total of 16 of the samples were positive for PV sequences. (C) Amplification of the Canine Papillomavirus E1 gene with the CP4 and CP5 primers. Ladder: Molecular Weight Marker, GeneRuler; H_2_O: negative control and C+: HeLa DNA (positive control); lane 1–21: biopsy samples. None of the analyzed biopsies was positive.

After DNA extraction, the analytical quality was confirmed by amplifying the β-globin gene (GH20 and PC04 primers, 285 bp) as well as the dog-specific Gliceraldehyde-3-phosphate dehydrogenase gene (dogGAPDHf and dogGAPDHr primers, 585 bp). A 76% of the samples (16 of 21) amplified for the β-globin gene and 95% (20 of 21) for the glyceraldehyde-3-phosphate dehydrogenase gene (Fig. 4). Afterward, a PCR was performed to amplify the viral L1 gene using MY09 and MY11 primers. The results demonstrate the presence of a 450 bp band in only seven samples (33%) of the 21 tested (Fig. 5A). In addition, a PCR was performed using the PVF and the FAP-64 primers which are used to detect several CPVs (Lange et al., 2011). The PC4 and PC5 pair of primers targeting the PV E1 gene were also included. The results obtained with the PVF/FAP64 primers showed a positivity of 76% (16 of 21) of the samples (Fig. 5B) whilst the amplification of E1 gene using the CP4 and CP5 primers, none of the analyzed samples were positive (Fig. 5C). PCR results are summarized in Table 4.

**Table 4:** Papillomavirus presence. Percentages of PV presence by each pair of primers used.

	*n* (%)
MY09/11	
Positive	6 (28.6)
Negative	15 (71.4)
CP4/5	
Positive	–
Negative	21 (100)
PVF/FAP64	
Positive	16 (76.2)
Negative	5 (23.8)

## Discussion

In this study were analyzed 21 patients clinically diagnosed with CTVT by cytology and histopathology according to Herrera, Thomas & Carlos (2006) at the Clinical Oncology Service of the civil association Esteriliza y Educa A.C. in Puerto Escondido, Oaxaca, Mexico.

A higher proportion of CTVT was found in females (67%) than in males (33%), which perhaps because in females the neoplasm occurs initially in the vestibule of the vagina and is more evident than in males. Also, in its initial form in males it can only be detected when the penis is drawn for copulation and it is when the owners perceive the presence of a new mass formation and ask for consultation. These results are in agreement with those reported by Ortega-Pacheco et al. (2003) that a higher prevalence of CTVT was reported in females than in males in a study conducted in Yucatán, Mexico. Moreover a retrospective analysis comprising 2,062 biopsies showed a 55% of CTVT (González-Chávez et al., 2015). Thus, the transmissible venereal tumor has a high prevalence and incidence among dog population.

Although some reports have shown an important proportion of patients with anemia at the moment of diagnosis (Herrera, Thomas & Carlos, 2006). The authors attributed the result to the fact that the tumors of the patients under study were ulcerated and bleeding, similar to those reported by Carvajal Santana et al. (2016) and Bianchi et al. (2011). It should also be mentioned that the patients analyzed in the present study did not present significant ulcerations. Since CTVT is an exposed neoplasia, it is susceptible for infection, thus the analysis of the presence of any bacterial infection was performed and no pathogenic bacteria were isolated from the tumor masses, probably because the patients were diagnosed 1-month latter after adoption and the owners of the patients included in the study revealed good hygienic practices.

In order to determine whether an infectious agent could be present in the CTVT, a series of PCR reactions were performed to detect PV sequences in the CTVT samples. After analyzing DNA integrity, all the patients were included. The results of the amplification of the L1 gene using the pair of universal primers MY11 and MY09 demonstrated the presence of viral DNA in 33% of the samples, these primers are widely used in molecular studies of detection of the Human PV (Gravitt et al., 2000) and its usefulness in the detection of animal viruses has also been reported in several studies in which CPV was identified in cutaneous pigmented plaques or in palmar lesions of Greyhound dogs (Luff et al., 2012; Anis et al., 2016).

Interestingly, when using the pair of primers PVF and FAP-64, targeting a region of the L1 gene encoding the major protein of the capsid highly conserved among PV, the percentage of positivity of the analyzed samples was 76%; however, the pair of primers CP4 and CP5 did not show positivity for any of the samples tested, this last pair of primers is designed to amplify a region of the E1 gene, which presents an important variation among the PV (Bergvall, Melendy & Archambault, 2013). This fact could partially explain the absence of E1 amplification in the analyzed samples. Since, PVF and FPA-64 primers have been used for the amplification of CPV sequences in a study by Lange et al. (2011), The results strongly suggest that the sequences detected in the samples belong to CPV, nevertheless, further studies are required to confirm the specific identity of the viral agent detected.

It is worth to mention that so far only 14 types been reported for CPVs assigned to three genera: Lambda, Tau and Chi, based on the nucleotide sequences of the L1 gene (De Villiers, 2013; Lange et al., 2013) and to date no specific detection method has been reported, being the study conducted by Lange et al. (2013) the only one that has provided PCR conditions that are capable of detecting CPV using the PVF and FPA-64 primers. Importantly, the results allow to suggest that the virus could be involved in the oncogenic process of the transmissible venereal tumor since a 17.8% of all cancer cases in humans are due to infectious agents, which include viruses (12.1%), bacteria (5.6%) and helminths (0.1%) (Plummer et al., 2016). Whether the virus was necessary for CTVT establishment remains an unresolved issue, and additional experiments are required to demonstrate the physical status of viral genome in CTVT cells such as in situ hybridization. Also, the possible role of the viral products in the maintenance of CTVT needs further exploration. It is worth to mention that seven viruses have been described as carcinogenic biological agents with the capacity to induce cellular transformation, either indirectly by chronic inflammation as a result of a continuous immune response to a persistent infection as its occurs with Hepatitis B and C viruses; or by direct action of its oncogenic proteins such as Human PV (Smith et al., 2016).

The presence of a viral agent in the pathogenesis of the CTVT has been previously studied (Kennedy, Yang & Allen, 1977), where pups and adult dogs were inoculated with CTVT tumor derived masses, after the incubation period transmission electron microscopy was performed to the resulting tumors searching for viruses. The authors declare the presence of virus-like particles in puppies derived samples, whilst in adult dogs no such particles were observed.

Cytological changes of cells infected with PV include acanthosis, prominent cytoplasmic vacuolization, nuclear atypia and binucleation. During a latent infection, the viral DNA remains inside the cell in an episomal form without replicating, therefore no identifiable morphological changes are observed in the cytology, so viral detection in this type of infections can only be done by molecular methods (De La Fuente-Villarreal et al., 2010). It is very likely that due to this fact (Kennedy, Yang & Allen, 1977) did not find viral structures in the analyzed samples, probably because that the adult patients have a stronger performance of the immune system or because the virus was latent or episomal without entering into a productive phase making it hard to detect unless using of more sensitive techniques such as PCR.

## Conclusions

A prevalence of DNA sequences of PV was found in the analyzed cases suggesting a possible role of this virus in the CTVT biology. Whether the virus is involved in tumor maintenance or how it was first involved in tumor establishment remains to be studied and further analyses will help to understand the probable contribution of this virus in the pathogeny of CTVT.

## Supplemental Information

10.7717/peerj.7962/supp-1 Supplemental Information 1 Mean values of Hemogram and biochemical values of patients. Each data is indicated as the average of the population. The biological reference value is indicated for each parameter.

10.7717/peerj.7962/supp-2 Supplemental Information 2 Complete uncropped gel for Figs. 5A and 5B.

10.7717/peerj.7962/supp-3 Supplemental Information 3 Complete uncropped gel for Fig. 5C.

## Additional Information and Declarations

### Data Availability

The following information was supplied regarding data availability: The uncropped gels of Fig. 5 are available as a Supplemental File.
