# Supplementary material for: Presence of Papillomavirus DNA sequences in the canine transmissible venereal tumor (CTVT)
Source: PeerJ. 2019 Oct 25;7:e7962. doi: 10.7717/peerj.7962 (PMC6816387; doi:10.7717/peerj.7962)
Supplement: Supplemental Information 1 — Each data is indicated as the average of the population. The biological reference value is indicated for each parameter. [file peerj-07-7962-s001.docx]

**Supplementary table 1. Mean values of Hemogram and biochemical values of patients.**

| Analyte | Population mean | Biological reference value |
| --- | --- | --- |
| Hemoglobin | 13.96 g/dL | 12.0-18.0 g/dL |
| Hematocrite | 41.55% | 37.0-55.0% |
| Erythrocytes | 5.96 x 106/μL | 5.5-8.5 x 106/μL |
| Globular Volume | 70.15 fL | 60-77 fL |
| Medium Globular Hemoglobin | 23.57 pg | 19.5-24.5 pg |
| Corpuscular Hemoglobin | 33.6 g/dL | 31.0-39.0g/dL |
| Reticulocytes | 25.1x10^3^/μL | <60x10^3^/μL |
| Total Leukocytes | 7317/μL | 6000-17000/μL |
| Total Neutrophils | 4727/μL | 3000-11800/μL |
| Segmented Neutrophils | 4541/μL | 3000-11500/μL |
| Neutrophils in band | 186/μL | 0-300/μL |
| Eosinophils | 442/μL | 100-1250/μL |
| Basophils | 33/μL | 0-100/μL |
| Monocytes | 183/μL | 150-1350/μL |
| Lymphocytes | 1929/μL | 1000-4800/μL |
| Platelets | 290x10^3^/μL | 160-430x10^3^/μL |
| Glucose | 4.06 mmol/L | 3.8-7.9 mmol/L |
| Urea | 6.82 mmol/L | 4.1-10.8 mmol/L |
| Ureic Nitrogen | 3.19 mmol/L | 1.9-5.0 mmol/L |
| Creatinine | 1.05 mg/dL | 0.5-1.6 mg/dL |
| Cholesterol | 3.48 mmol/L | 1.81-3.88 mmol/L |
| Triglycerides | 88 mg/dL | 29-291.0 mg/dL |
| Uric Acid | 0.65 mg/dL | 0.40-0.70 mg/dL |
