# Supplementary figures and images for: Presence of Papillomavirus DNA sequences in the canine transmissible venereal tumor (CTVT)
Source: PeerJ. 2019 Oct 25;7:e7962. doi: 10.7717/peerj.7962 (PMC6816387; doi:10.7717/peerj.7962)

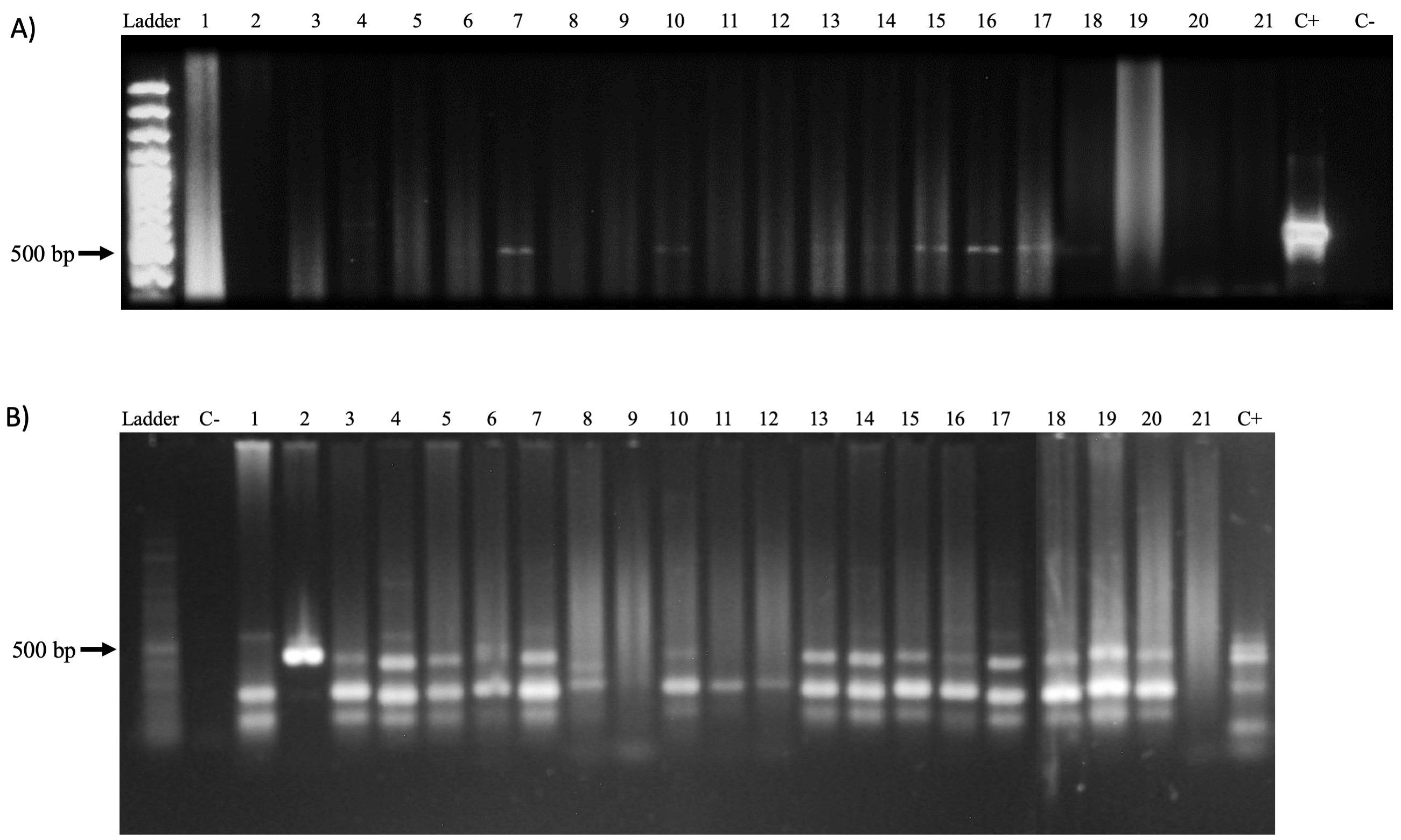

Supplement: Supplemental Information 2 [file peerj-07-7962-s002.jpeg]

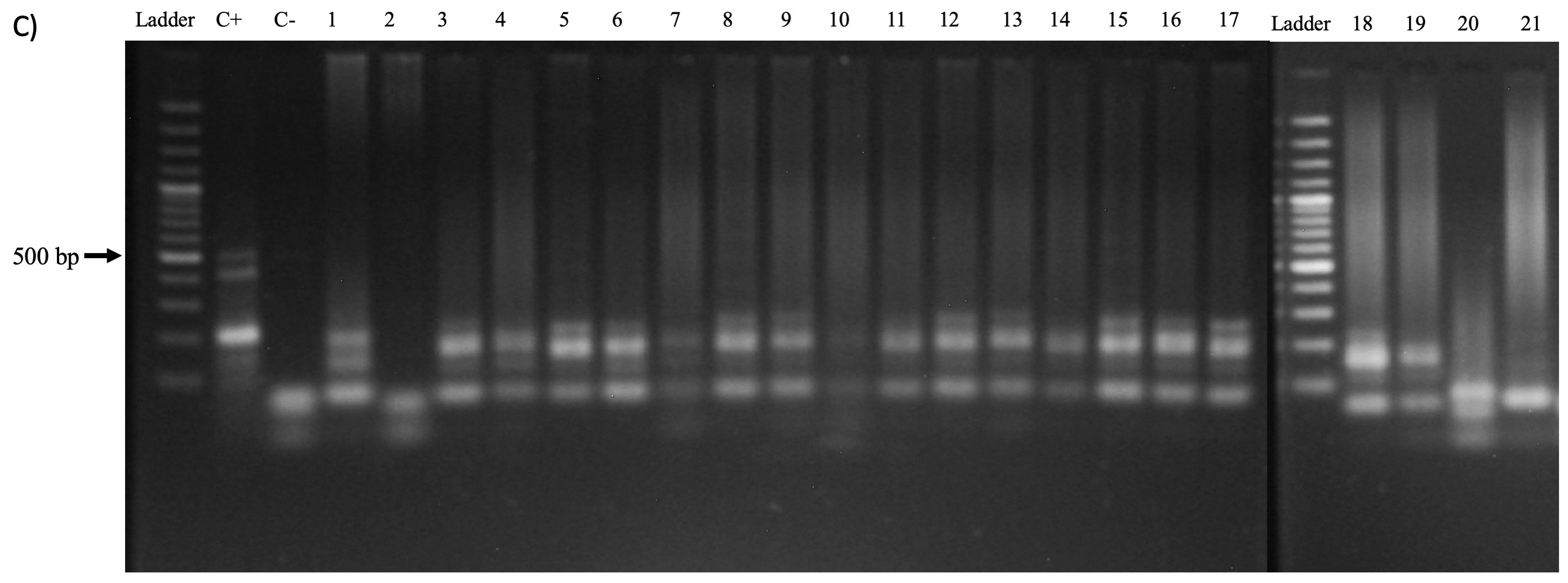

Supplement: Supplemental Information 3 [file peerj-07-7962-s003.jpeg]
